# Protective role of N-acetylcysteine and Sulodexide on endothelial cells exposed on patients’ serum after SARS-CoV-2 infection

**DOI:** 10.3389/fcimb.2023.1268016

**Published:** 2023-12-21

**Authors:** Justyna Rajewska-Tabor, Patrycja Sosińska-Zawierucha, Malgorzata Pyda, Maciej Lesiak, Andrzej Bręborowicz

**Affiliations:** ^1^ I Clinic of Cardiology, Unit of Magnetic Resonance, Poznan University of Medical Sciences, Poznan, Poland; ^2^ Department of Pathophysiology, Poznan University of Medical Sciences, Poznan, Poland; ^3^ Collegium Medicum, Zielona Góra, Poland

**Keywords:** COVID-19, endothelial cells, N-acetylcysteine, Sulodexide, endotheliitis

## Abstract

Severe acute respiratory syndrome coronavirus-2 causes hyperinflammation and activation of coagulation cascade and, as a result, aggravates endothelial cell dysfunction. N-acetylcysteine and Sulodexide have been found to mitigate endothelial damage. The influence on coronary artery endothelial cells of serum collected after 4 ± 1 months from coronavirus infection was studied. The concentrations of serum samples of interleukin 6, von Willebrand Factor, tissue Plasminogen Activator, and Plasminogen Activator Inhibitor-1 were studied. The cultures with serum of patients after coronavirus infection were incubated with N-acetylcysteine and Sulodexide to estimate their potential protective role. The blood inflammatory parameters were increased in the group of cultures incubated with serum from patients after coronavirus infection. Supplementation of the serum from patients after coronavirus infection with N-acetylcysteine or Sulodexide reduced the synthesis of interleukin 6 and von Willebrand Factor. No changes in the synthesis of tissue Plasminogen Activator were observed. N-acetylcysteine reduced the synthesis of Plasminogen Activator Inhibitor-1. N-acetylcysteine and Sulodexide increased the tPA/PAI-1 ratio. N-acetylcysteine may have a role in reducing the myocardial injury occurring in the post-COVID-19 syndrome. Sulodexide can also play a protective role in post-COVID-19 patients.

## Introduction

Severe acute respiratory syndrome coronavirus-2 (SARS-CoV-2) is a pandemic disease affecting the respiratory system and also other organs of the human body (Raman et al., 2021). The virus invades the cells of the respiratory system, entering it mainly through endothelial cells, and then attacks other organs and endothelial cells themselves (Bonaventura et al., 2021). The action of the virus and the human defense mechanisms activate anti-viral processes, causing hyperinflammation, which then activates neutrophils, monocytes, and platelets and results in the activation of the coagulation cascade, possibly leading to intravascular thrombosis (Bermejo-Martin et al., 2020; Evans et al., 2020; Teuwen et al., 2020).

At the cellular level, the structural and functional dysfunction of endothelium is due to the lack of nitric oxide (NO), cellular oxidative stress, the inflammatory process, and a damaged glycocalyx structure. Myocarditis is one of the complications of COVID-19, which may aggravate endothelial cell dysfunction (Bonaventura et al., 2021). All the above and the resulting myocardial damage, acting on the endothelial cells of the coronary artery (Fogarty et al., 2021; Yin et al., 2021; Bogdanov and Khirmanov, 2022), may be responsible for the long-COVID-19 syndrome, which affects 10%–30% of patients (Davis et al., 2021; Ceban et al., 2022).

N-acetylcysteine (NAC) and Sulodexide have been found to mitigate endothelial damage and dysfunction (Ciszewicz et al., 2007; Sosińska-Zawierucha et al., 2018; Sosinska-Zawierucha et al., 2019). By suppressing the secretion of pro-inflammatory cytokines such as NF-κB, IL-8, and IL-6, NAC reduces the chemotactic migration of monocytes (Geiler et al., 2010; Zhang et al., 2018). NAC has also been shown to have a protective role in reducing the replication of various viruses, including the human immunodeficiency virus (HIV) (ho and Douglas, 1992) or the respiratory syncytial virus (RSV) (Mata et al., 2012). This protective effect of NAC on endothelial cells reduces the adverse effects of viruses on the vascular endothelium (Jin et al., 2020). The results of earlier studies of other RNA viruses have suggested that NAC might also have a similar role in SARS-CoV-2 infection. NAC has also been shown to have the potential ability to inhibit SARS-CoV-2 replication (Ibrahim et al., 2020; Nasi et al., 2020; Poe and Corn, 2020; du Preez et al., 2022b). Also, the supportive effect of Sulodexide in the acute phase of COVID-19 has been demonstrated in the literature (Gonzalez-Ochoa et al., 2021; Bednarz et al., 2022; Melkumyants et al., 2022). The long-term protective effect of these substances is still being investigated (Charfeddine et al., 2022).

We present results from the study in which the effect of serum isolated from the post-COVID-19 patients on the function of the human coronary endothelial cells was studied in *in vitro* culture. Additionally, we evaluated if NAC and Sulodexide modify the post-COVID-19 serum-induced changes in these cells.

## Materials and methods

The effect of serum on the coronary endothelial cells collected from 12 patients with prior positive PCR tests for SARS-CoV-2 infection but otherwise healthy was evaluated in an *in vitro* culture. Only the patients with mild symptoms were included in the study. None of them was hospitalized during infection and had no need of oxygen treatment. They were isolated for at least 10 days with symptomatic treatment only. The laboratory tests taken after 4 ± 1 months did not show any abnormalities: all patients had normal levels of D-dimers, C-reactive protein, creatinine, and leukocytes.

No patient showed a sign of the acute or persisted myocardial injury, as reflected by troponin I and NT-proBNP serum concentration. All patients had undergone a mild form of COVID-19, manifesting itself mainly in fever (9; 75%), headache (8; 67%), fatigue (7; 58%), and muscle pain (7; 68%).

The serum of 12 healthy individuals with no past COVID-19 experience was used as the control. The protocol of the study was approved by the Bioethical Committee of the Poznan University of Medical Sciences. All patients gave their written consent to participate in the study.

The biochemical characteristics of the studied groups are shown in **Table 1**. The concentrations of serum samples of interleukin 6 (IL-6), the von Willebrand Factor (vWF), the tissue Plasminogen Activator (tPA), and Plasminogen Activator Inhibitor-1 (PAI-1) taken after 4 ± 1 months after patients’ recovery were studied with the commercially available ELISA kits (R&D Systems, Minneapolis, MN, USA).

**Table 1:** Characteristic of studied group and control group.

	Control	Post-COVID-19 group	*p*
Number of patients	12	12	ns
Age (years)	42.8 ± 7.3	41.9 ± 10.7	ns
Time after COVID-19 (months)	0	4.2 ± 1.1	<0.001
Blood IL-6 (pg/mL)	3.5 ± 1.3	45.6 ± 90.9	<0.001
Blood vWF (μg/mL)	5.6 ± 0.9	41.1 ± 6.6	<0.001
t-PA (mg/mL)	3.7 ± 1.5	34.8 ± 16.6	<0.001
PAI-1 (ng/mL)	18.9 ± 2.6	74.1 ± 13.3	<0.001

IL-6, interleukin 6; vWF, von Willebrand Factor; t-PA, tissue Plasminogen Activator; PAI-1, Plasminogen Activator Inhibitor-1, ns - not significant. Correlation revised with Spearman tests.

### *In vitro* culture of the endothelial cells

During the experiments, the primary cultures of human coronary artery endothelial cells (CAEC) obtained from Cell Applications, Inc. (San Diego, California, USA) were used. The culture for the growth of the cells was provided by the producer. The cells were grown to monolayers in 75-cm^2^ culture flasks, and were subsequently harvested with trypsin 0.05%–EDTA 0.02% solution and seeded into the 48-well culture plates. Experiments were performed on the endothelial monolayers.

### Effect of the serum samples on the endothelial cells

The endothelial monolayers in 48-well culture plates were exposed for 24 h to the standard culture medium supplemented with the serum sample (20%v/v). In our earlier experiments, we found that such treatment did not induce any morphological changes in the endothelial cells and while the MTT test (Abcam, Cambridge, UK) was used, it did not reduce viability either. The cells were exposed to MTT salt [3-(4,5-dimethylthiazol-2-yl)-2,5-diphenyltetrazolium bromide] for 3 h at 37°C. The generated formazan product was lysed and the lysate absorbance was measured at 595 nm.

The following experimental groups were studied, with cells exposed to the following solutions:

1. Culture medium (Control),
2. Culture medium supplemented with serum (20% v/v) from healthy donors,
3. Culture medium supplemented with serum (20% v/v) from the post-COVID-19 patients,
4. Culture medium supplemented with serum (20% v/v) from the post-COVID-19 patients + N-Acetylcysteine 1 mmol/L, and
5. Culture medium supplemented with serum (20% v/v) from the post-COVID-19 patients + Sulodexide (0.5 LRU/mL).

### The cell parameters studied

#### Intracellular oxidative stress

At the end of the 24-h incubation, in six wells from each group, the oxidative stress was measured. Free radicals generated within the cells were measured during their 45-min incubation at 37°C with a 2’7’dochlorodihydrofluorescein diacetate probe. After the lysis of the cells, the fluorescence of the cells lysates was measured in a fluorimeter at a wavelength of 485 nm for excitation and 535 nm for emission. The number of free radicals generated was expressed as a number of cells counted in six wells from each group, in separate wells.

#### Secretory activity of the cells

After 24 h of incubation, the medium in all wells was replaced with the standard culture medium for evaluation of the cell’s secretory activity in the following 24 h. At the end of the incubation, the medium was collected from all wells, spun down (200*g*; 10 min) and frozen at −86°C for further analysis. The cells were harvested with a trypsin 0.05%–EDTA 0.02% solution and counted in a hemocytometer. In the supernatants, the concentrations of molecules IL-6, tPA, PAI-1, and vWF were measured with the standard ELISA kits (R&D Systems, Minneapolis, MN, USA). The secretion of the molecules from the endothelial cells was expressed per number of cells.

#### Statistical analysis

The results are presented as a mean ± SD. The statistical analysis was performed with the Mann–Whitney test, or ANOVA with the *post hoc* analysis of the Kruskal-Wallis test. The correlation between the studied groups was measured with the Spearman test. A *p*-value less than 0.05 was considered statistically significant.

## Results

There was a significant difference between the blood inflammatory parameters, which were increased in the post-COVID-19 group compared to the control group (**Table 1**). The exposure of CAEC to the sera used in the experiments significantly modified the functional properties of the cells.

**Figure 1:**
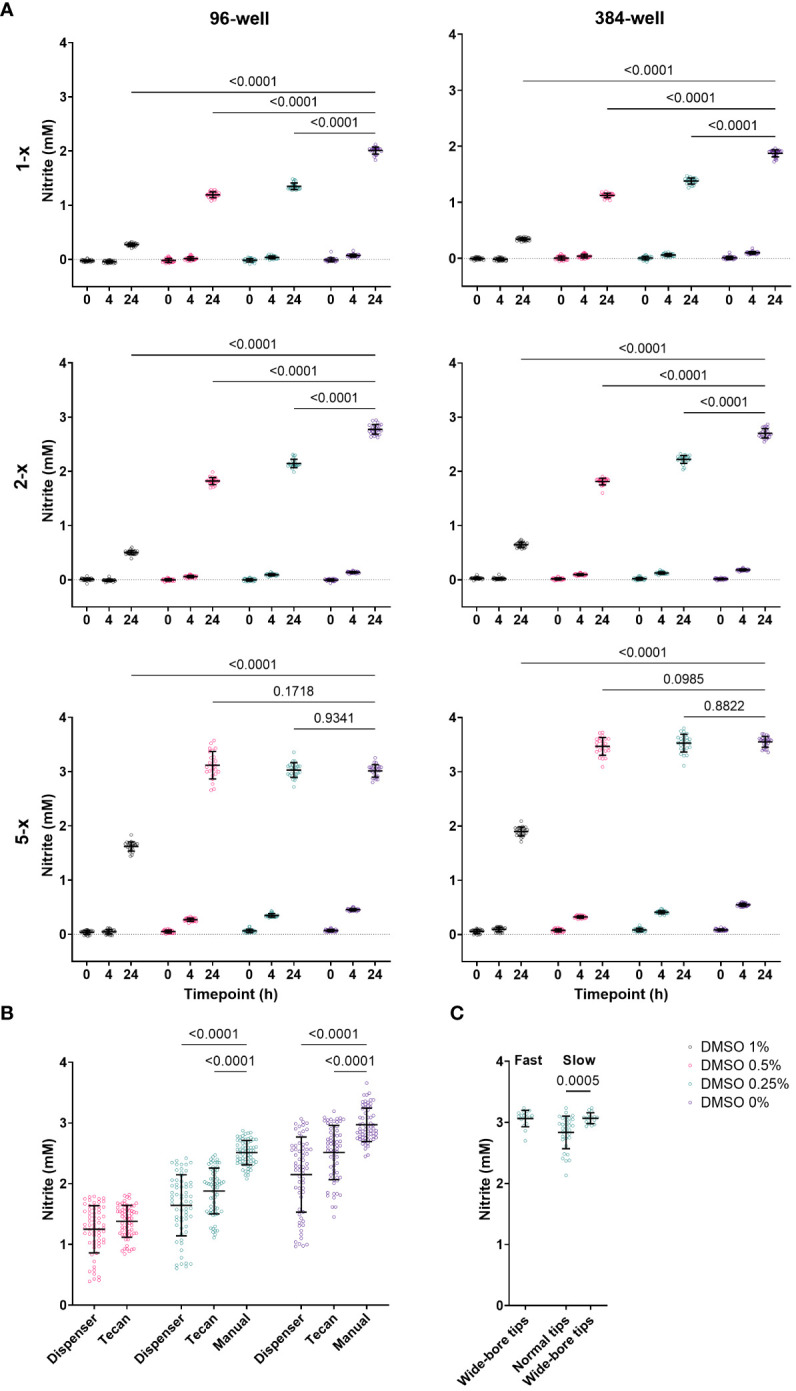
Intracellular generation of free radicals in CAEC exposed to culture medium (Medium), culture medium supplemented with 20% control serum (Control), 20% Post-COVID-19-serum (Post-COVID), 20% Post-COVID-19 serum supplemented with N-Acetylcysteine 1 mmol/L (Post-COVID+NAC), or Post-COVID-19 serum with Sulodexide 0.5 LRU/mL (Post-COVID+Sul).

**Figure 2:**
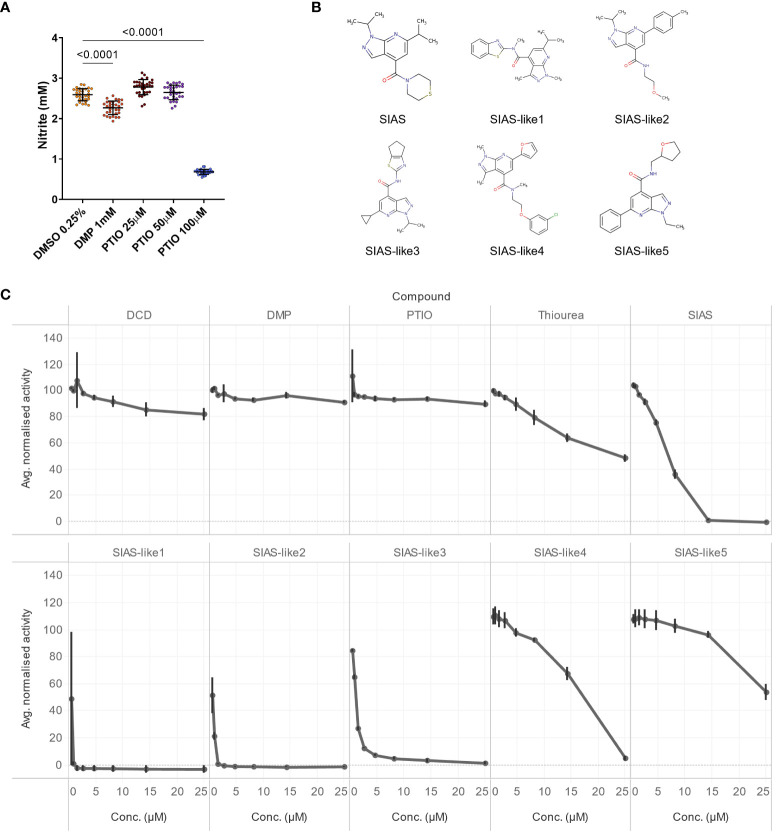
Synthesis of IL-6 **(A)** and vWF **(B)** in CAEC exposed to culture medium (Medium), culture medium supplemented with 20% control serum (Control), 20% Post-COVID-19 serum (Post-COVID), 20% Post-COVID-19 serum supplemented with N-Acetylcysteine 1 mmol/L (Post-COVID+NAC), or Post-COVID-19 serum with Sulodexide 0.5 LRU/mL (Post-COVID+Sul).

**Figure 3:**
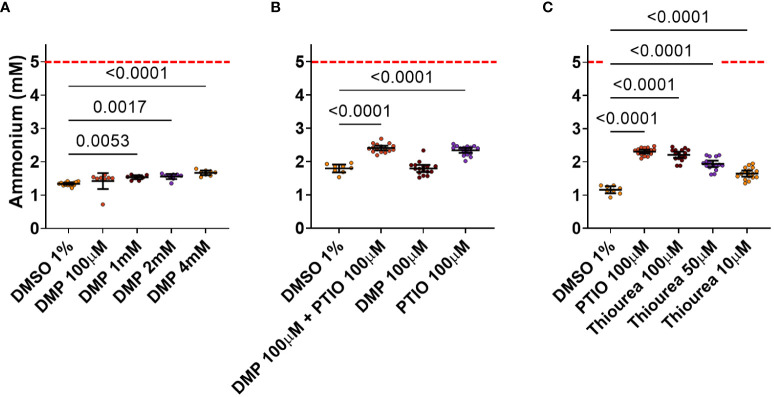
Synthesis of tPA **(A)** and PAI-1 **(B)** in CAEC exposed to culture medium (Medium), culture medium supplemented with 20% control serum (Control), 20% Post-COVID-19 serum (Post-COVID), 20% Post-COVID-19 serum supplemented with N-Acetylcysteine 1 mmol/L (Post-COVID+NAC),or Post-COVID-19 serum with Sulodexide 0.5 LRU/mL (Post-COVID+Sul).

In the presence of the post-COVID-19 serum, the intracellular generation of free radicals was increased (+29%, *p* < 0.001) in comparison with the cells exposed to the control medium (**Figure 1**). Supplementation of the post-COVID-19 serum with NAC reduced the intracellular oxidative stress (−23%, *p* < 0.001). Post-COVID-19 serum stimulated the synthesis of IL-6 in CAEC (+43%, *p* < 0.001) as compared to cells treated with the control serum (**Figure 1A**). Supplementation of the post-COVID-19 serum with NAC or Sulodexide reduced the synthesis of IL-6 to −18%, *p* < 0.02 and −24%, *p* < 0.01, respectively (**Figure 1A**). In CAEC exposed to the post-COVID-19 serum, the synthesis of vWF increased by 22%, *p* < 0.01, as compared to the control serum (**Figure 1B**). NAC used as a supplement to the post-COVID-19 serum reduced the synthesis of vWF by 30%, *p* < 0.001 (**Figure 2**). No changes in the synthesis of tPA in CAEC treated with the post-COVID-19 serum were observed (**Figure 3A**). However, the synthesis of PAI-1 was increased in the endothelial cells exposed to the post-COVID-19 serum (+20%, *p* < 0.01) (**Figure 3B**). NAC reduced the stimulatory effect of the post-COVID-19 serum on the synthesis of PAI-1 in CAEC: 17%, *p* < 0.002 (**Figure 3B**). The tPA/PAI-1 ratio, reflecting the net fibrinolytic activity of serum, was reduced in the post-COVID-19 group as compared to the control serum: 1.3 ± 0.1 vs. 1.6 ± 0.1 (*p* < 0.001). NAC and Sulodexide increased the tPA/PAI-1 ratio in the cells treated with the post-COVID-19 serum to 1.6 ± 0.1 (*p* < 0.01) and 1.5 ± 0.1 (*p* < 0.05), respectively.

## Discussion

The post-COVID-19 syndrome and its long-term effects, in particular, are still poorly understood and affect many areas and organs, showing a broad spectrum of symptoms. An injury of endothelial cells acquired during COVID-19 may have long-lasting consequences. Haffke et al. have proved that the serum markers of an endothelial injury may be elevated as late as 8 months after COVID-19 or even later (Haffke et al., 2022). In this study, we have proved that the inflammation of endothelial cells in SARS-CoV-2 infection may not only be caused directly by the virus but also develop much later since the serum of the convalescents retains the possibility of inducing inflammation even months after infection. The exposure of CAEC to post-COVID-19 serum generates oxidant stress and stimulates CAEC to produce pro-inflammatory cytokines: IL-6, vWF, tP, and PAI-1.

The degree of sulfation of heparan sulfate in the glycocalyx can have the influence in susceptibility to SARS-CoV-2 infection (Clausen et al., 2020; Liu et al., 2021; du Preez et al., 2022a; du Preez et al., 2022b). SARS-CoV-2 infection oxigenative stress and proteolysis and therefore it has been demonstrated to cause rapid depletion of sulfur amino acids (Bourgonje et al., 2021; du Preez et al., 2022b). Undersulfation of glycocalyx can also influence the response to the infection. Sulfur donors, like NAC and Sulodexide, could play a beneficial role in the endothelial cells’ injury. NAC treatment has been demonstrated to successfully replenish sulfur amino acids within hours following NAC supplementation during acute phase of infection (Bourgonje et al., 2021). Our study also demonstrates the decrease of the levels of endothelial dysfunction biomarkers, which can prove the potential protective role of sulfur donors months from COVID-19.

The thromboembolic complications of COVID-19 can be monitored by comparing the elevated levels of D-dimers, the fibrinogen, and the vWF with the normal ranges of PT, aPTT, and platelet count (Helms et al., 2020; Iba et al., 2020). As reported in the literature, endothelial dysfunction biomarkers such as vWF and PAI-1 are increased in COVID-19 patients compared to healthy subjects. They seem to have a prognostic significance, being associated with more severe forms of the disease and a high mortality (Goshua et al., 2020; Rovas et al., 2021). However, it has also been proved that sometimes thromboembolic complications develop in COVID-19 patients with normal PT, aPTT, aPTT, and platelet results. In such an event, they may be monitored by checking the D-dimer, fibrinogen, or vWF levels. Fogarty et al. showed sustained endotheliopathy measured by vWF level at a median of 68 days following SARS‐CoV‐2 infection (Fogarty et al., 2021). This experiment revealed that there still occurs endothelial damage and increased secretion of endothelial factors such as vWF and PAI-1 4 months after infection.

It has been reported that NAC decreases the binding of the virus to cells, decreases virus replication, has anti-inflammatory and antioxidant activity, and modulates the immune system (Mohanty et al., 2021; Wong et al., 2021; du Preez et al., 2022b). Therefore, NAC is used in treating the acute period of SARS-CoV-2 infection, mainly intended to combat the cytokine storm (Mohanty et al., 2021; Wong et al., 2021).

Our experiment demonstrated the protective role of NAC for coronary artery endothelial cells. When exposed to post-COVID-19 serum and treated with NAC, CAEC released lower levels of pro-inflammatory cytokines than when exposed to post-COVID-19 serum without the NAC treatment. Moreover, we also proved that the incubation of endothelial cells with the serum of post-COVID-19 patients and supplemented with NAC resulted in the reduction of the secretion of the tested substances to the level of the CEAC secretion of the serum of healthy subjects.

Sulodexide is another possible candidate for application in COVID-19 therapy, particularly in patients with a mild form of the disease (Charfeddine et al., 2022; Melkumyants et al., 2022). Sulodexide is a mixture of glycosaminoglycans consisting of 20% of dermatan sulfate and 80% fast-moving heparin. Its *in vitro* effects are comparable to enoxaparine, at least in anti-hemostatic effects (Coccheri and Mannello, 2013). Sulodexide produces multifaceted effects: by increasing tPA production and inhibiting platelet aggregation, it activates arterial and venous anticoagulant and fibrinolytic processes, and it shows an anti-inflammatory activity, including the inhibition of IL-6 production (Coccheri and Mannello, 2013; Sosinska-Zawierucha et al., 2019). It may be applied to treat different types of endothelial cells, as has already been demonstrated in other studies (Sosińska-Zawierucha et al., 2018; Sosinska-Zawierucha et al., 2019). Essentially, Sulodexide may also be used in patients with renal impairment and is less likely associated with bleeding risk and heparin-induced thrombocytopenia.

In studies by Gonzalez-Ochoa et al., when compared to a placebo, Sulodexide has been found to possibly reduce the risk of hospitalization and the need for oxygen supply while improving laboratory parameters without increasing the risk of bleeding in early high-risk COVID-19 patients (Gonzalez-Ochoa et al., 2021). In this experiment, we have demonstrated that Sulodexide inhibits IL-6 secretion from endothelial cells at a later stage of COVID-19. The effects on other factors that we also tested are less significant. Despite this tendency to decrease the secretion of tPA, vWF, and PAI, no statistically significant decrease in these factors has been determined. This finding, however, requires further investigation.

In conclusion, a risk of a potential injury of endothelial cells remains months after COVID-19. NAC may have a role in reducing the myocardial injury occurring in the post-COVID-19 syndrome by reducing the endothelial injury of coronary arteries. Likewise, Sulodexide may also play a particular role in protecting endothelial cells in patients with or after COVID-19 infection.

## Data availability statement

The raw data supporting the conclusions of this article will be made available by the authors, without undue reservation.

## Ethics statement

The studies involving humans were approved by Bioethics committee in Poznan University of Medical Sciences, Poznan, Poland. The studies were conducted in accordance with the local legislation and institutional requirements. The participants provided their written informed consent to participate in this study.

## Author contributions

JR-T: Data curation, Formal analysis, Investigation, Project administration, Resources, Writing – original draft, Writing – review & editing, Supervision. PS-Z: Formal analysis, Investigation, Methodology, Software, Validation, Writing – review & editing. MP: Conceptualization, Supervision, Validation, Writing – review & editing. ML: Supervision, Funding acquisition, Writing – review & editing. AB: Conceptualization, Funding acquisition, Investigation, Methodology, Project administration, Supervision, Writing – review & editing.
